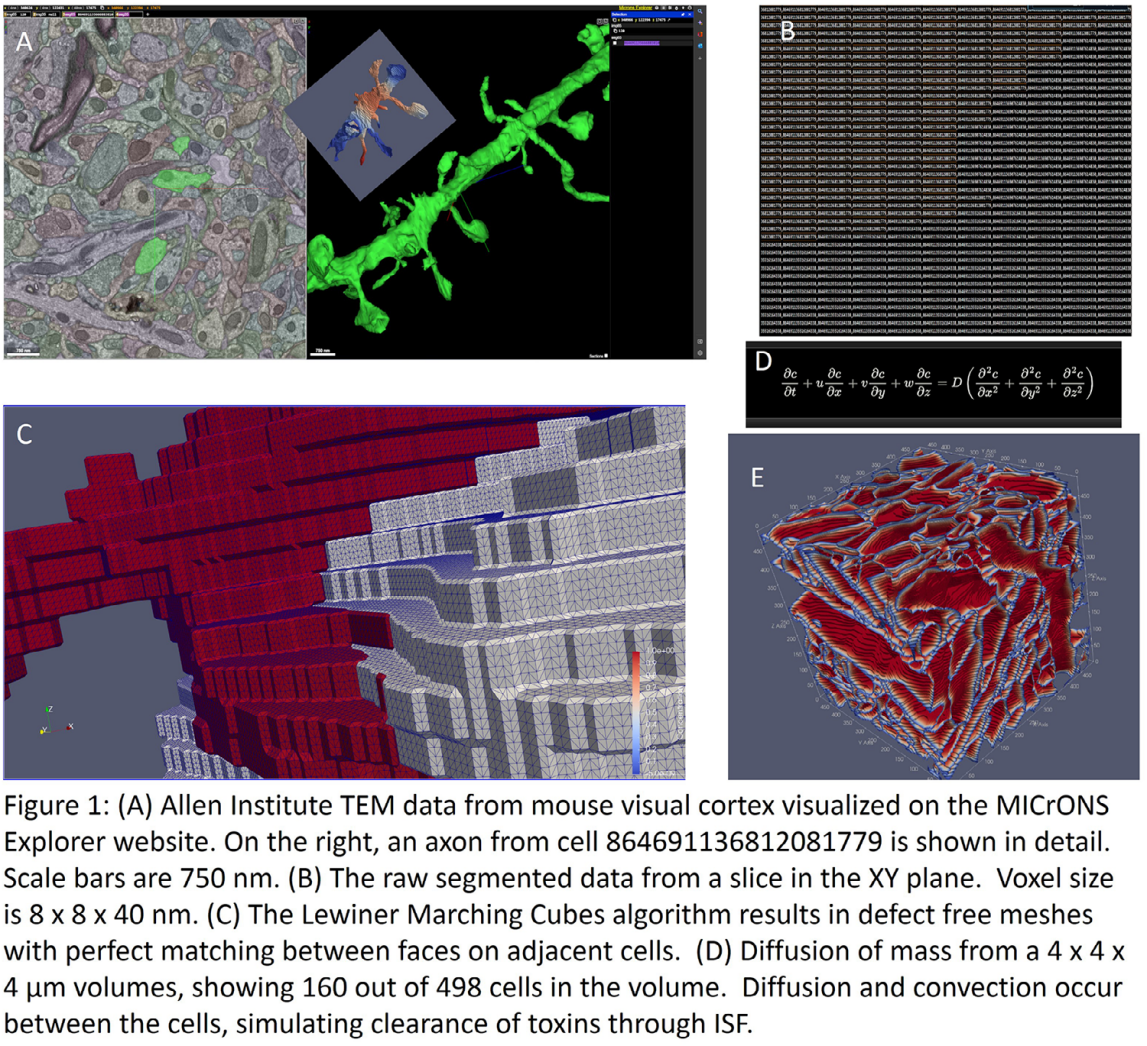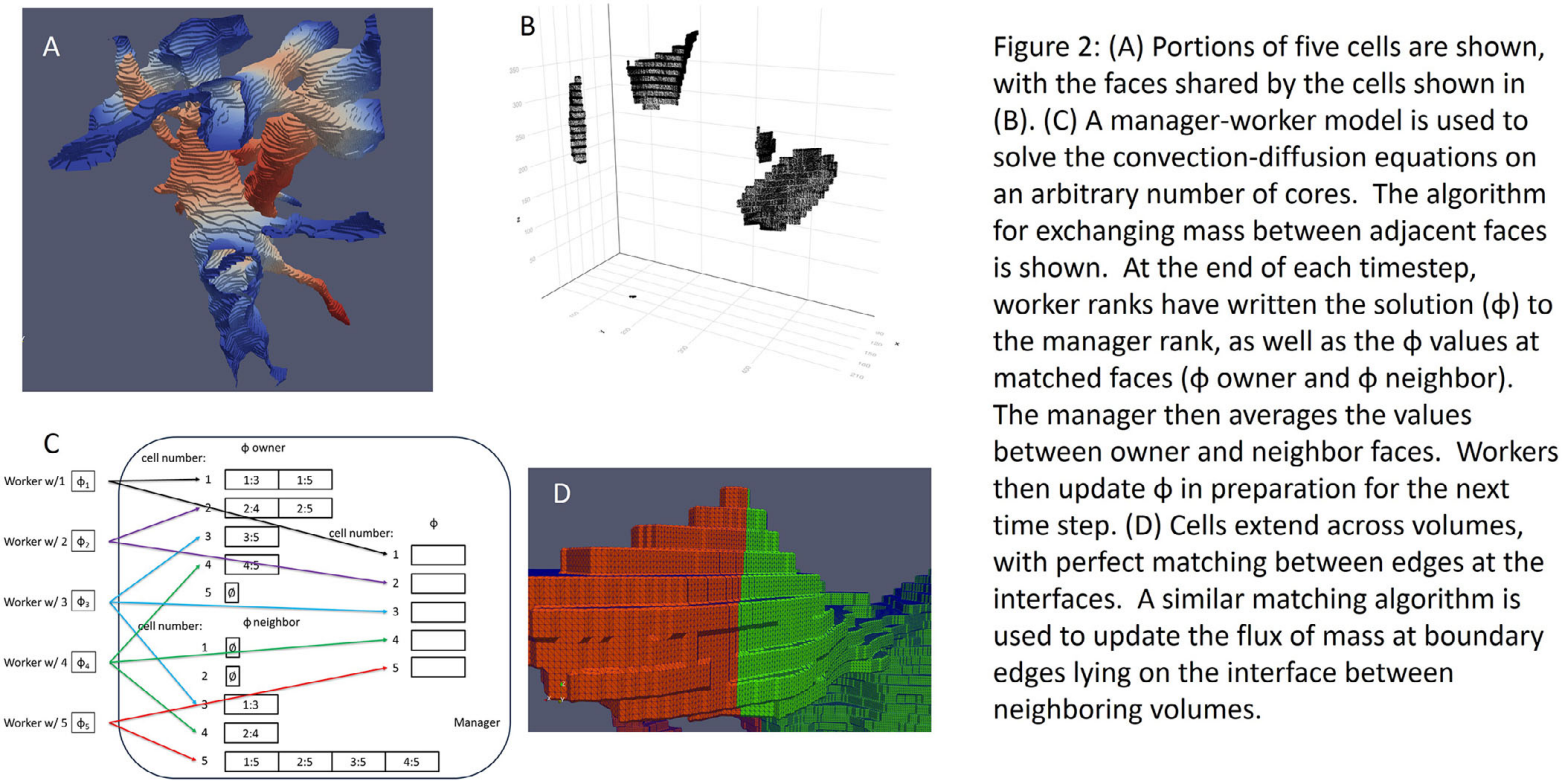# A new pipeline for converting neuroimaging data into computational models of ISF/CSF processes

**DOI:** 10.1002/alz.091362

**Published:** 2025-01-03

**Authors:** Donald L. Elbert

**Affiliations:** ^1^ University of Washington, Seattle, WA USA

## Abstract

**Background:**

A multitude of high‐quality imaging modalities exist that provide structural data at unprecedented levels of detail. Tissue ultrastructure greatly influences the rate of transport of proteins and other molecules that contribute to neurodegeneration. However, our ability to model flow and diffusion processes in the brain lags behind the quality of the neuroimaging data. Here, a new framework will be presented for converting neuroimaging data into computational fluid dynamics (CFD) models in a scalable manner suitable for high performance computing. TEM data from the Allen Institute will illustrate its capabilities.

**Methods:**

Segmented TEM images were downloaded from the MicronsExplorer public storage cache. Segmented image stack volumes (4 × 4 × 4 µm) were converted into surface meshes using the Lewiner Marching Cubes algorithm. Convection and diffusion along the surface of individual cells was modeled. After each timestep mass was allowed to exchange between neighboring cells, implemented on a high performance computing cluster using the MPI protocol on 40 or more computing cores. Multiple volumes were analyzed simultaneously, with updates of the flux between cells in different volumes occurring at each time step. These steps were performed with new custom CFD code written in C and Julia.

**Results:**

Segmented z‐stacks of TEM data from the MICrONS Explorer website were downloaded in 4 × 4 × 4 µm blocks, which typically contain ≈500 distinct cells (Fig. 1A&B). The meshing algorithm leads to perfect matching between adjacent cells (Fig. 1C). The convection‐diffusion equations are solved, modeling flow/diffusion of solutes between cells (Fig. 1D&E). Equations are solved for all ≈500 distinct cells in parallel, with concentrations at matching faces re‐balanced at each time step (Fig. 2A&B). A manager‐worker MPI model allows for solution on an arbitrary number of cores (Fig. 2C). The interfaces between cell surfaces across volumes are also perfectly matched, allowing for adjustment of the flux of mass between volumes at each timestep (Fig. 2D).

**Conclusions:**

A new modeling framework is described for solution of transport equations on high performance computing clusters for any imaging modality that produces segmented z‐stacked images.